# Supplementary material for: Phylogenomic coupling of F1 chemosensory and archaellum systems across archaea and monoderm bacteria
Source: ISME Commun. 2026 Nov 3;6(1):ycag288. doi: 10.1093/ismeco/ycag288 (PMC13747009; doi:10.1093/ismeco/ycag288)
Supplement: Supplementary file 1 [file supplementary_materials_ycag288.zip › Supplementary_materials_ycag288.docx]

**Supplementary data legends:**

**Supplementary Figure 1: Distribution of archaellum genes and F1-type chemosensory systems across prokaryotes, illustrating their occurrence across bacterial and archaeal lineages.** Maximum likelihood phylogeny was constructed using FastTree v2 from a concatenated alignment of two conserved marker proteins (COG0051: RpsJ and COG0186: RpsQ), comprising 6,308 positions, for the 22k NCBI curated reference bacterial and archaeal genomes. The tree is rooted using the archaeal clade. From the inner to the outer rings, the annotation layers show: phylum-level taxonomy; the number of archaellum genes detected in each genome, shown in red; the number of flagellar genes detected in each genome, shown in green; and the count of F1-type chemosensory components (CheA), shown in blue. All organism details have been provided in the Supplementary Table 1A. Major phylum names have been provided for key representative phyla and organism names have been provided for a few discussed organisms for better understanding.

**Supplementary Figure 2: Maximum-likelihood phylogeny of core archaellum and chemosensory proteins.** The tree was reconstructed using FastTree v2 from a concatenated alignment of ArlI (arCOG01817), ArlJ (arCOG01809) and F1-type CheA sequences. Individual protein sequences were aligned using MAFFT, and the concatenated alignment was trimmed with TrimAl using the gappyout parameter, resulting in a final alignment of 550 positions. The inner ring indicates phylum-level taxonomy, while the outer ring represents class-level classification of the organisms.

**Supplementary Table 1:**

**1A.** Distribution of motility and chemosensory systems across 22,000 bacterial and archaeal genomes. Genes encoding archaellum, bacterial flagellum, T4aP and Tad systems were identified using MacSyFinder v2, while CheA homologues and associated chemosensory signalling modules were detected using hmmscan of HMMER suite 3.4 against the Pfam database v38 along with curated CSS class profiles. Archaeal and bacterial genomes are shaded yellow and purple respectively, to aid visual separation.

**1B.** Chemosensory system profiles stratified by the presence of archaellum genes across ~22,000 prokaryotic genomes. The analysis distinguishes F1-type and ACF-type signalling modules and compares their occurrence in organisms that encode archaellum components versus those that do not.

**1C.** Distribution of F1-type and ACF-type chemosensory modules among 226 distinct Chloroflexota genomes that contain archaellum genes. These genomes correspond to 244 organisms described in Sivabalasarma et al.; redundant entries were removed to generate a unique set for comparative analysis.

**1D.** Representative examples from the set of 226 Chloroflexota archaellum-encoding genomes, illustrating the genomic neighbourhoods surrounding archaellum loci and their proximity to F1-type chemosensory systems. These cases highlight the possibility of F1 CSS coordinating and interacting with archaellum machinery.

**1E.** The archaeal dataset is presented with the detection status of the three core genes, arlI, arlJ and F1-type cheA. Organisms from the reference and Chloroflexota datasets that encode all three genes are also included here.

**1F.** Comprehensive listing of all F1-type CheA proteins recovered from the dataset and assigned to the three major clades identified in the maximum-likelihood phylogeny. For each entry, the table provides the phylogenetic clade designation, genome accession with locus tag and annotated protein description, phylum-level taxonomy, and broad cell-envelope classification as Archaea, monoderm or diderm.
