# Supplementary material for: Phylogenomic coupling of F1 chemosensory and archaellum systems across archaea and monoderm bacteria
Source: ISME Commun. 2026 Nov 3;6(1):ycag288. doi: 10.1093/ismeco/ycag288 (PMC13747009; doi:10.1093/ismeco/ycag288)
Supplement: Supplementary file 1 [file supplementary_materials_ycag288.zip › Supplementary_materials_ycag288_Figure-2.pdf]

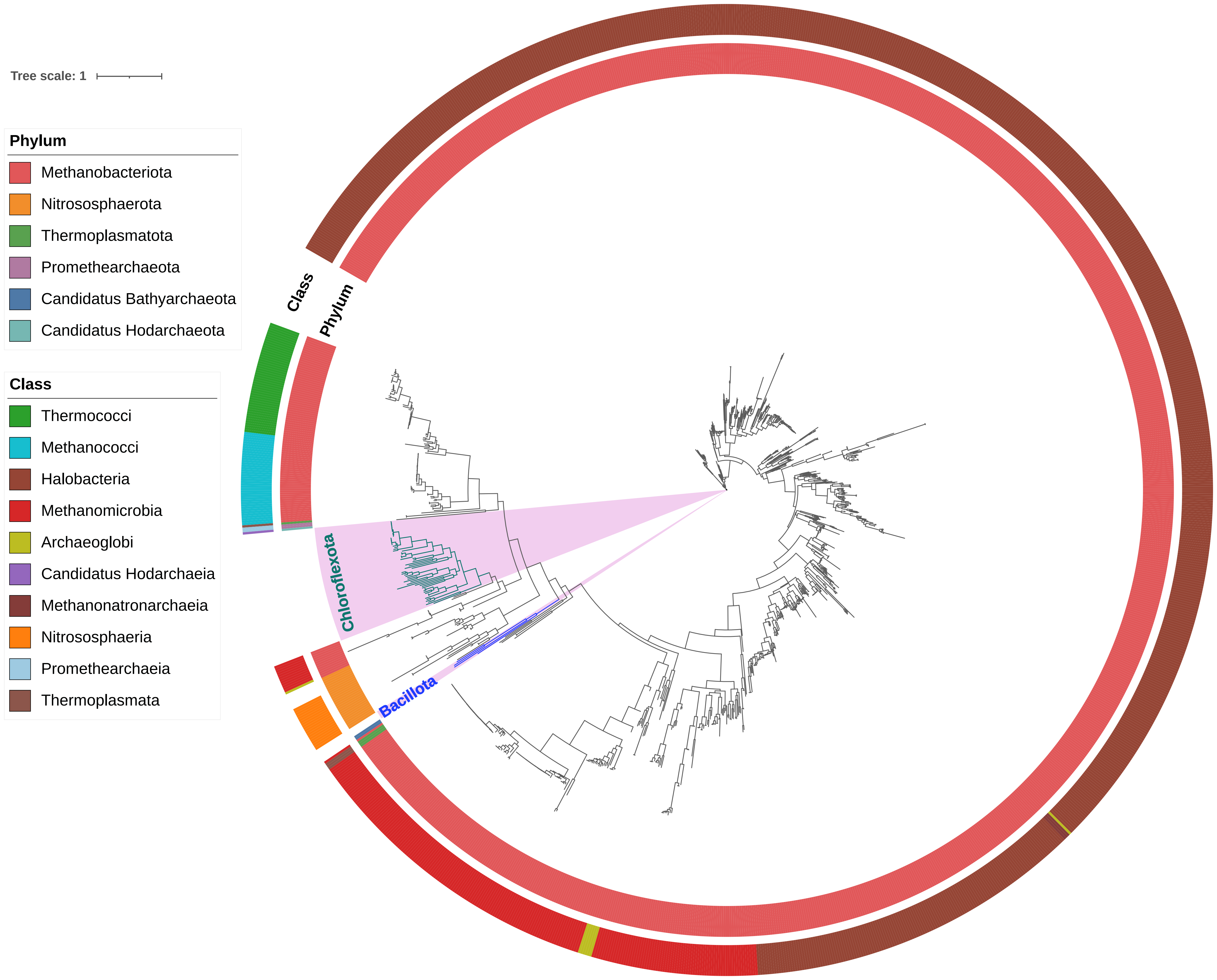

Tree scale: 1
Phylum
Methanobacteriota
Nitrososphaerota
Thermoplasmatota
Promethearchaeota
Candidatus Bathyarchaeota
Candidatus Hodarchaeota
Class
Thermococci
Methanococci
Halobacteria
Methanomicrobia
Archaeoglobi
Candidatus Hodarchaeia
Methanonatronarchaeia
Nitrososphaeria
Promethearchaeia
Thermoplasmata
Class
Phylum
Chloroflexota
Bacillota
