# Supplementary material for: Phylogenomic coupling of F1 chemosensory and archaellum systems across archaea and monoderm bacteria
Source: ISME Commun. 2026 Nov 3;6(1):ycag288. doi: 10.1093/ismeco/ycag288 (PMC13747009; doi:10.1093/ismeco/ycag288)
Supplement: Supplementary file 1 [file supplementary_materials_ycag288.zip › Supplementary_materials_ycag288_Figure-1.pdf]

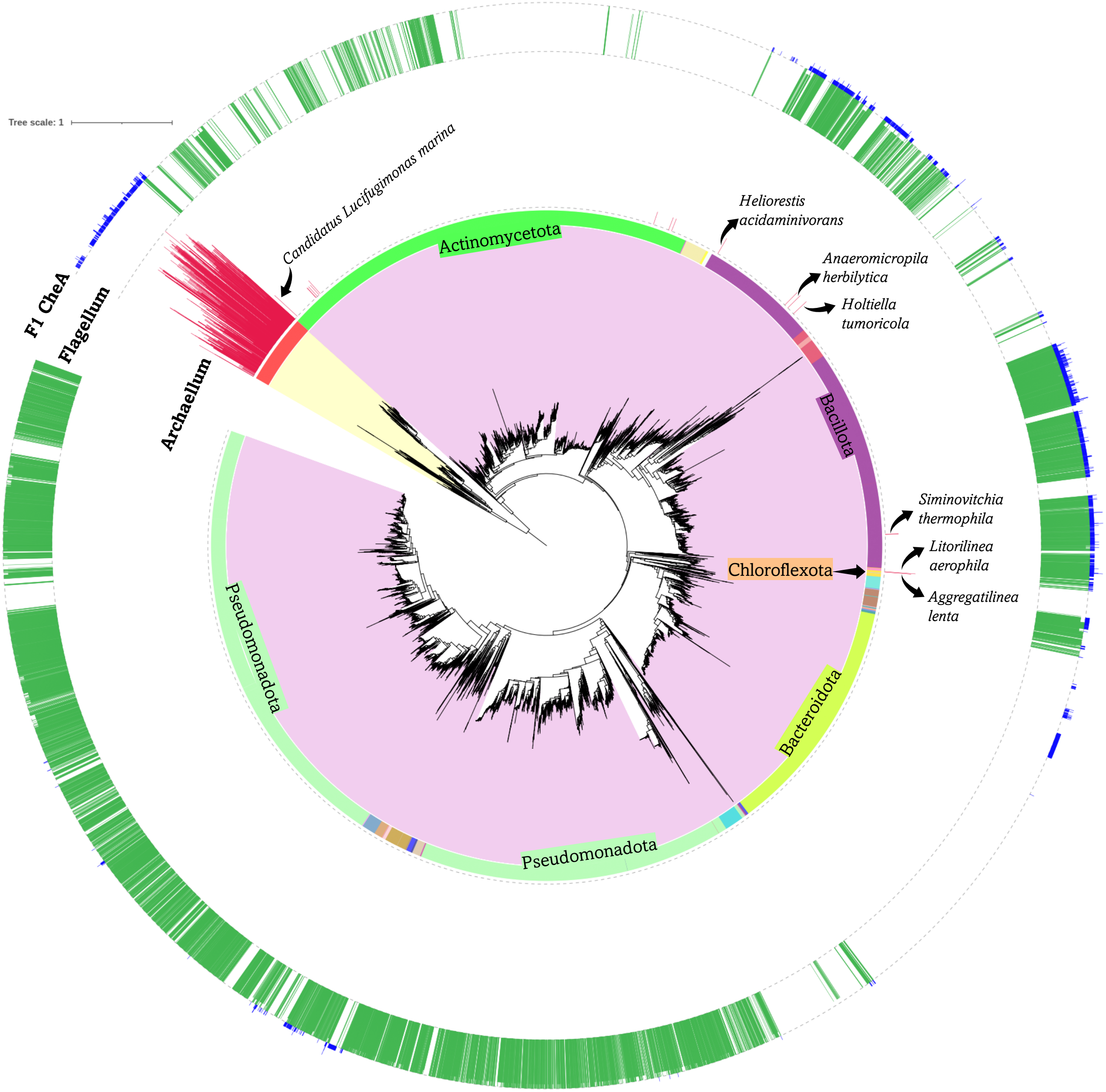

Tree scale: 1
F1 CheA
Flagellum
Archaellum
Candidatus Lucifugimonas marina
Actinomycetota
Heliorestis acidaminivorans
Anaeromicropila herbilytica
Holtiella tumoricola
Bacillota
Siminovitchia thermophila
Litorilinea aerophila
Aggregatilinea lenta
Chloroflexota
Bacteroidota
Pseudomonadota
Pseudomonadota
